# Relationship between cerebral oxygenation, cardiac output, and blood pressure during transitional period in extremely low gestational age neonates

**DOI:** 10.3389/fped.2023.1187769

**Published:** 2023-08-15

**Authors:** Poorva Deshpande, Caio Barbosa de Olivera, Amish Jain, Cecil Hahn, Prakesh S. Shah, Anne-Marie Guerguerian, Patrick J. McNamara

**Affiliations:** ^1^ Department of Pediatrics, Mount Sinai Hospital, Toronto, ON, Canada; ^2^ Department of Pediatrics, University of Toronto, Toronto, ON, Canada; ^3^ Neurosciences and Mental Health, Hospital for Sick Children, Toronto, ON, Canada; ^4^ Department of Pediatrics, University of Iowa, Iowa, IA, United States

**Keywords:** cerebral oxygenation, cardiac output, transition, preterm, extremely low gestational age

## Abstract

**Objective:**

To describe the relationship between cerebral oxygenation, cardiac output, arterial blood pressure (BP), and cerebral blood flow velocity in extremely low gestational age neonates (ELGANs) during transition.

**Methods:**

This study comprises secondary analyses from a prospective observational study conducted at a tertiary Neonatal Intensive Care Unit. Recruited ELGANs underwent cerebral saturation (CrSO_2_) monitoring and serial echocardiography during 72 h from birth. Correlative analyses of CrSO_2_ and cerebral fractional tissue oxygen extraction (CFTOE) with left (LVO) and right ventricular output (RVO), superior vena cava (SVC) flow, middle cerebral artery blood flow mean velocity (MCA.MV), systolic (SBP), diastolic (DBP), and mean (MBP) BP were conducted.

**Results:**

Fifty ELGANs with median (range) gestational age of 25.9 (23.1–27.9) weeks were recruited. Echocardiography was performed sequentially at a median (range) age 5.0 (3.8–6.6), 17.3 (15.4–19.4), 31.0 (27.0–34.1), and 53.7 (49.3–58.3) hours. RVO, LVO, CrSO_2_, and SBP increased over time but no changes in MBP, DBP, CFTOE, MCA.MV or SVC flow were noted. A weak correlation was identified between CrSO_2_ and SBP (*r*^2 ^= 0.11, *p *= 0.047) and MBP (*r*^2^ = 0.12, *p *= 0.04) at 17.3 (15.4–19.4) hours. No correlation of either CrSO_2_ or CFTOE with any measures of blood flow was identified.

**Conclusion:**

There is a weak correlation between measures of cardiac output, BP, and MCA.MV with both CrSO_2_ and CFTOE in ELGANs during transition. Whether this finding suggests intact cerebral autoregulation requires prospective evaluation in a cohort of sick ELGANs.

## 1. Introduction

Maintaining optimal cerebral blood flow during transition is crucial for extremely low gestational age neonates (ELGANs), due to a high risk of brain injury during this period. Historically, clinicians in neonatal intensive care units (NICUs) have relied on arterial blood pressure (BP) as a surrogate marker of end-organ perfusion, including cerebral perfusion. BP has been shown to correlate poorly with left ventricular output (LVO) measured on echocardiography (1). Further, therapies targeted at maintaining arbitrary BP values have not proven to reduce brain injury (2). As BP alone is an unreliable measure of hemodynamic stability and provides limited diagnostic insight on the etiology or the impact on tissue perfusion, there has been a move towards using newer modalities such as targeted neonatal echocardiography (TNE) and prefrontal cerebral near-infrared spectroscopy (NIRS) to provide a comprehensive hemodynamic evaluation (3). From a clinical perspective, an integrative approach by combining information obtained from multiple modalities is likely to provide a more holistic appraisal of cardiovascular and brain health. Increasingly, over the last decade, TNE is being used in many NICUs across the world for the assessment of cardiac function and outputs; however, its widespread use is limited by a lack of round-the-clock availability of personnel trained in imaging and interpretation. Although cerebral NIRS is now being adopted by a handful of NICUs for continuous real-time monitoring of cerebral oxygenation and perfusion, it is not yet the standard of care for cerebral monitoring in preterm infants during periods of vulnerability such as during postnatal transition. The standard of care in most centers is to rely on arbitrary, and unproven, measures of BP (4). Even in centers with hemodynamic programs providing advanced echocardiography measures and enhanced diagnostic precision, there may be no longitudinal neurophysiologic surveillance which is a gap in practice given the vulnerability of the immature brain. While the practical application of multi-modal monitoring in NICUs is still evolving, the knowledge of how cerebral blood flow is affected by changes in systemic hemodynamics is important but remains poorly investigated. Whether BP or echocardiographic markers of systemic blood flow correlate with cerebral oxygenation is not clearly understood with conflicting findings in the literature (5–11). Therefore, the primary aim of this study was to describe the relationship of cerebral oxygenation with cardiac output, BP, and cerebral Doppler measurements in ELGANs during the first 72 h after birth. We hypothesized that an increase in cerebral oxygenation during the transitional period is linearly correlated with cardiac output and superior vena cava (SVC) flow but not with BP.

## 2. Materials and methods

### 2.1. Study design and setting

This study includes secondary analyses of data collected from a prospective observational cohort study investigating the feasibility of multimodal monitoring in ELGANs (12), and was conducted at the tertiary NICU of Mount Sinai Hospital, Toronto. The study was approved by the Institutional Research Ethics Board. A sample size of 50 patients was decided *a priori* based on feasibility (12). Parental consent was obtained either prior to or within 6 h of birth. Inborn and outborn infants with gestational age (GA) at birth between 23^+0^ and 27^+6^ weeks were eligible for inclusion. Infants with known genetic or chromosomal abnormalities, congenital heart defects, and hematological disorders were excluded. Infants who received chest compressions or cardiac medications at birth were included. An indwelling umbilical arterial catheter was inserted for invasive BP monitoring in infants weighing <750 g or if deemed suitable by the clinical team. All infants were managed using an intraventricular hemorrhage (IVH) prevention bundle for the first 72 h. The care bundle included supine neutral midline head position with upper body elevation at 15 degrees, minimal routine handling limited to every 4–6 h, avoidance of hypocarbia below partial pressure of carbon dioxide of 40 mmHg, and avoidance of rapid fluid boluses. A routine brain ultrasound was performed between postnatal days 4 and 7.

### 2.2. Echocardiography and cerebral prefrontal NIRS

After initial stabilization in the resuscitation room, pre-frontal cerebral NIRS monitoring was commenced using the INVOS 5100C Cerebral Oximeter, (Medtronic, Minneapolis, USA) and continued until 72 h from birth. Neonatal sensors were applied over Mepitel dressing on the right side of the infant's forehead as per standard recommendations on the use of cerebral NIRS sensors in neonates (13). Cerebral saturation (CrSO_2_) values displayed on the monitor screen were covered by an opaque screen to maintain blinding from the clinical team. Echocardiography and brain ultrasound were performed sequentially at 4–8 h, 12–18 h, 24–36 h, and 48–60 h using the Vivid E9 cardiovascular ultrasound system (GE Healthcare, Wisconsin, USA) with the 12 MHz multi-frequency neonatal transducer. Imaging was timed with routine nursing handling time performed by trained operators (PD, AJ, DIR, or SB), all of whom had received prior standardized TNE training. Guidelines from the American Society of Echocardiography for TNE were applied for standardized views, image acquisition, measurements, and safety (14).

LVO, right ventricular output (RVO), and SVC flow were calculated according to the formulae below:

$$\begin{array}{rl} \mathrm{LVO}(\mathrm{ml}/\min/\mathrm{kg}) & =[\pi [\mathrm{Left}\;\mathrm{ventricular}\;\mathrm{outflow}\;\mathrm{tract}\;(\mathrm{LVOT})\\ & \;\;\;\;\;\mathrm{Diameter}/2]{}^{2}\;\times \;\mathrm{LVOT}\;\mathrm{velocity}\;\mathrm{time}\;\mathrm{integral}\;\\ & \;\;\;\;(\mathrm{VTI})\;\times \;\mathrm{heart}\;\mathrm{rate}\;\left(\mathrm{HR}\right)]÷\mathrm{Body}\;\mathrm{weight} \end{array}$$

$$\begin{array}{rl} \mathrm{RVO}(\mathrm{ml}/\min/\mathrm{kg}) & \;=\;\{\pi [\mathrm{right}\;\mathrm{ventricular}\;\mathrm{outflow}\;\mathrm{tract}\;(\mathrm{RVOT})\\ & {\;\;\;\;\;\;\;\;\mathrm{diameter}/2]}^{2}\times \mathrm{RVOT}\;\;\;\mathrm{VTI}\;\;\times \mathrm{HR}\}\;÷\;\mathrm{Body}\;\mathrm{weight} \end{array}.$$

$$\begin{array}{rl} \mathrm{SVC}\;\mathrm{flow}\;(\mathrm{ml}/\min/\mathrm{kg}) & =[\pi {\left(\mathrm{SVC}\;\;\;\mathrm{diameter}/2\right)}^{2}\\ & \;\;\;\;\;\times \mathrm{SVC}\;\;\;\mathrm{VTI}\times \mathrm{HR}]\;÷\mathrm{Body}\;\mathrm{weight} \end{array}.$$

The middle cerebral artery (MCA) was visualized in the cross-sectional plane by placing the transducer at the lateral coronal suture and maximal mean velocity was measured with pulsed Doppler using a 12 MHz multi-frequency neonatal transducer.

### 2.3. Clinical hemodynamic management

Prophylactic indomethacin was given to infants born <25 weeks GA and/or who weighed <750 g at birth, according to a standardized unit protocol. Research echocardiography findings were disclosed to the clinical team either upon request or if any of the following findings were noted: severe myocardial dysfunction (subjective impression), suspicion of congenital cardiac disease, malpositioned central catheter tip, or large PDA with diameter >2.5 mm. These criteria were based on prior consensus amongst the practicing neonatologists in our unit. The decision to initiate pharmacotherapy to modulate a hemodynamically significant PDA was at the attending clinician's discretion. Our unit did not follow any specific policy for early PDA screening or treatment during the study period. Whenever pharmacotherapy was used, indomethacin was the first-line medication. The management of hypotension was also at the discretion of the medical team. A TNE consultation for hypotension or pulmonary hypertension was obtained upon the medical team's request.

### 2.4. Data acquisition and management

Relevant data on maternal and perinatal characteristics, delivery details, ventilation at birth, and cardiovascular support during the first 72 h were collected. CrSO_2_ was monitored continuously. Cerebral fractional tissue oxygen extraction (CFTOE) was calculated according to the formula CFTOE = [Preductal Oxygen saturation (SpO_2_) − CrSO_2_]/SpO_2_. Echocardiography images were stored in the hospital's archive. A trained research echocardiography technician (ME), who was blinded to the clinical data performed all measurements and calculations offline using the Echopac software Version 11 (GE Healthcare, Wisconsin, USA).

### 2.5. Definitions

IVH was classified as Grades I–IV according to the Papille grading classification (15), based on the latest brain ultrasound between days 4 and 7. Brain ultrasound was reported by a radiologist as part of clinical reporting. Clinical stability during the first postnatal week was defined as the absence of the following: IVH Grade III/IV, culture-positive early-onset sepsis, acute pulmonary hypertension (aPH) defined as hypoxic respiratory failure requiring >50% fraction of inspired oxygen, with either clinical or echocardiographic signs elevated pulmonary pressures and treated with inhaled nitric oxide, spontaneous intestinal perforation (SIP), hypotension treated with volume and/or inotropic agents or vasopressors and mortality irrespective of the cause. PDA was considered moderate-to-large when the diameter was >1.5 mm. Patent foramen ovale (PFO) was considered significant when the diameter was >2.0 mm. LVO was defined as low when below 150 ml/min/kg. Low SVC was defined as below 30 ml/min/kg.

### 2.6. Statistical analysis

All statistical analyses were performed using R Version 3.6.2 (R Core Team, 2019). Data were described using parametric and non-parametric tests, as appropriate. CrSO_2_ and CFTOE values for every subject were averaged for 10 min prior to the start of imaging. Arterial BP (averaged over 10 min prior to imaging) or non-invasive BP (single recording using oscillometry within one hour of the onset of imaging) were used for analysis. The relationships of CrSO_2_ and CFTOE with LVO, RVO, SVC flow, systolic, diastolic, and mean BP (SBP, DBP and MBP, respectively) and middle cerebral artery blood flow mean velocity (MCA.MV) were tested using Pearson's correlation. Subgroup correlative analyses were performed on the following groups: clinically stable infants, after the exclusion of infants with PDA diameter >1.5 mm and infants with LVO <150 ml/min/kg. The statistical significance of changes in serial measurements was assessed using repeated measures analysis of variance (ANOVA).

## 3. Results

Fifty ELGANs with a median (range) gestational age (GA) of 25.9 (23.1–27.9) weeks and birthweight of 795 (660–875) grams were recruited (Table 1). Two infants received chest compressions at birth, none received epinephrine. Of these, one infant was classified as ‘clinically stable’ and the other as’ clinically unstable’ because of aPH. Thirty-nine infants (78%) had a clinically stable course in the first week and the remaining 11 patients were classified as unstable; specific co-existing pathologies were as follows: IVH Grade III/IV (*n* = 4, of which one infant died due to Grade IV IVH and one developed SIP); aPH (*n *= 4); SIP (*n *= 2, of which one infant with IVH Grade III); mortality due to septic shock related to late onset sepsis on day 4 of life (*n *= 1). None of the infants had culture-positive early-onset sepsis.

**Table 1:** Perinatal and neonatal characteristics (*N* = 50).

Gestational age (weeks)	25.9 (23.1–27.9)			
Female sex	20 (40)			
Birth weight (grams)	795.0 (660.0–875.0)			
Maternal age (years)	32.0 (27.0–35.0)			
Antenatal steroids	2 doses	1 dose	None 5 (10)	
	38 (76)	7 (14)		
Maternal chorioamnionitis on placental histopathology	29 (58)			
Preterm prolonged rupture of membranes	27 (54)			
Intrapartum magnesium sulphate	38 (76)			
Delayed cord clamping	28 (56)			
Vaginal delivery	23 (46)			
Apgar				
1 min	4.0 (0–9)			
5 min	7.5 (1−10)			
Umbilical arterial pH	7.25 (6.81−7.49)			
Initial respiratory support	Conventional ventilation	Continuous positive airway pressure	High frequency oscillation	
	15 (30)	18 (36)	17 (34)	
Prophylactic indomethacin	21 (42)			
Hemoglobin (Hb) values over 72 h	0–12 h	12–36 h	36–60 h	60–72 h
Age (hours)	1.20 (0.50–11.95)	24.65 (12.15–35.70)	48.07 (36.62–58.70)	63.65 (61.27–71.77)
Hb (Hb g/dl)	148.0 (89.0–218.0)	141.5 (105.0–192.0)	131.0)89.0–172.0)	125.0 (93.0–159.0)
Partial pressure of carbon dioxide (pCO_2_) over 72 h	0–12 h	12–36 h	36–60 h	60–72 h
Age (hours)	2.92 (0.53–11.68)	24.55 (12.25–35.95)	46.77 (36.13–59.88)	64.28 (60.07–72)
pCO_2_ (mmHg)	46 (21–76)	42 (19–68)	44 (21–74)	45 (26–84)

Data are described as *n* (%) or median (range) as appropriate.

### 3.1. Temporal hemodynamic changes

CrSO_2_ monitoring was commenced at a median (range) age of 4.9 (2.2–12) hours and median (range) duration of recording was 63 (46.7–69.2) hours. Arterial BP was available for 86% of the measurements, the remainder were obtained via oscillometry. Echocardiography was performed at median (range) age of 5.0 (3.8–6.6), 17.3 (15.4–19.4), 31 (27.0–34.1), and 53.7 (49.3–58.3) hours. RVO, LVO, CrSO_2_, and SBP increased over time, but significant changes in MBP, DBP, CFTOE or MCA.MV or SVC flow were not noted (Table 2). Forty-two (84%), 28 (56%), 31 (62%), and 25 (50%) infants had a PDA at the 4 respective time points. The median diameter of PDA is shown in Table 2. Of these, 14, 3, 6, and 8 infants, at the 4 time points respectively, had a PDA diameter >1.5 mm. At the 4 respective time points, 24 (48%), 16 (32%), 8 (16%), and 6 (12%) infants had LVO lower than 150 ml/min/kg. Only one infant had low SVC flow each at the first and last scan. None of the infants in the cohort received treatment for systemic hypotension in the first 72 h. One infant developed hypotension on day 4 (outside of the transitional period) due to septic shock and was treated with dopamine and dobutamine. Four infants had aPH and were treated with inhaled nitric oxide initiated at 20 ppm. Only one infant received intravenous milrinone during the first 72 h for management of aPH.

**Table 2:** Hemodynamic and echocardiographic variables.

Age (hours) [median (IQR)]	**5.02** **(3.8–6.6)**	**17.3** **(15.4–19.4)**	**31** **(27–34.1)**	**53.7** **(49.3–58.3)**	*p*
LVO (ml/min/kg)	146.74 (83.54–300.57	163.11 (82.27–255.73)	179.48 (63.79–264.32)**	196.90 (113.60–293.10)***	0.017
RVO (ml/min/kg)	140.22 (54.82–330.22)	161.44 (63.07–245.42)	171.36 (89.41–341.31)***	193.81 (91.02–316.34)***	<0.0001
SVC flow (ml/min/kg)	74.29 (23.47–173.33)	77.42 (43.13–270.37)	91.54 (45.11–161.69)	85.47 (39.97–201.53)	0.8
MCA.MV (m/s)	0.11 (0.06–0.18)	0.12 (0.07–0.23)	0.13 (0.07–0.29)	0.13 (0.08–0.29)	0.38
CrSO_2_ (%)	71.04 (59.09–84.78)	73.77 (52.45–83.98)	74.15 (57.83–86.18)	69.81 (55.88–86.29)	0.03
CFTOE	0.26 (0.11–0.39)	0.22 (0.00–0.93)	0.22 (0.07–0.39)	0.26 (0.06–0.81)	0.36
MBP (mmHg)	34.50 (23.00–65.00)	38.00 (25.00–48.00)	38.00 (29.00–68.00)	38.00 (30.00−54.00)	0.5
SBP (mmHg)	42.00 (30.00−72.00)	46.00 (33.00−69.00)	47.50 (34.00−76.00)*	49.00 (36.00−72.00)**	0.002
DBP (mmHg)	30.00 (17.00−61.00)	32.00 (21.00−42.00)	33.00 (24.00−64.00)	31.00 (22.00–46.00)	0.27
Heart rate (beats/min)	157.00 (126.00−183.00)	152.00 (114.00–191.00)	155.00 (124.00–182.00)	160.00 (135.00–186.00)	0.57
PDA Diameter^a^ (mm)	1.35 (0.50–2.97)	1.05 (0.33–2.14)*	1.18 (0.35–3.50)	1.12 (0.50−3.10)	0.004
Number of infants with PDA > 1.5 mm (*n*)	14 (0.28)	3 (0.06)	6 (0.12)	8 (0.16)	–
Number of infants with PFO > 2.0 mm (*n*)	12 (0.24)	7 (0.14)	12 (0.24)	7 (0.14)	–

Data are presented as median (range) or *n* (%). LVO, left ventricular output; RVO, right ventricular output; SVC, superior vena cava; MCA.MV, middle cerebral artery mean velocity; SBP, systolic blood pressure; DBP, diastolic blood pressure; MBP, mean blood pressure; PDA, patent ductus arteriosus^a^ (when present); PFO, patent foramen ovale.

Results are expressed as median (range) or frequency (percentage). Changes over time were studied using repeated measures analysis of variance (ANOVA). Pairwise comparisons were studied using Tukey HSD test; CFTOE, cerebral fractional tissue oxygen extraction; CrSO_2_, cerebral saturation.

* *p* < 0.05.

** *p* < 0.01.

*** *p* < 0.001 compared to baseline.

### 3.2. Correlative analyses

Overall, no correlation between CrSO_2_ and CFTOE and any of RVO, LVO, MCA.MV, SBP, DBP, MBP, and SVC flow were noted at all time points. A very weak, correlation between CrSO_2_ and both SBP [*r*^2 ^= 0.11, (*p *= 0.047)] and MBP [*r*^2 ^= 0.12, (*p *= 0.04)] at 17.3 (15.4–19.4) hours (Table 3) was identified. This correlation was no longer statistically significant when analyses were restricted to measurements of BP obtained via an indwelling umbilical arterial catheter (Table 3). In the subgroup of patients deemed stable, a moderate correlation between DBP and both CrSO_2_ [*r*^2^ = 0.5, (*p *= 0.01)] and CFTOE [*r*^2^ = 0.37, (*p *= 0.046)] and a weak-moderate correlation between MBP and CrSO_2_ [*r*^2 ^= 0.39, (*p *= 0.038)] was noted at the time of first evaluation. After the exclusion of observations with PDA diameter >1.5 mm and PFO >2.0 mm, no significant correlation between RVO and LVO and CrSO_2_ or CFTOE at any time-point were noted. There was no correlation of CrSO_2_ or CFTOE with LVO in the subgroup of infants with LVO <150 ml/min/kg.

**Table 3:** Correlations between CrSO_2_ and CFTOE with cardiac output, MCA.MV, blood pressure.

Age (hours) [median (IQR)]		5.02 (3.8–6.6)		17.3 (15.4–19.4)		31 (27–34.1)		53.7 (49.3–58.3)	
LVO	CrSO_2_	0.023		0.002		0.003		0.029	
	CFTOE	0.005		0.005		0.005		0.001	
RVO	CrSO_2_	0.006		0.002		0.000		0.036	
	CFTOE	0.006		0.058		0.005		0.017	
MCA.MV	CrSO_2_	0.002		0.006		0.048		0.007	
	CFTOE	0.000		0.000		0.081		0.023	
SVC flow	CrSO_2_	0.009		0.000		0.022		0.006	
	CFTOE	0.000		0.000		0.057		0.028	
			Arterial catheter only		Arterial catheter only		Arterial catheter only		Arterial catheter only
SBP	CrSO_2_	0.012	0.009	0.109*	0.032	0.017	0.049	0.036	0.007
	CFTOE	0.004	0.002	0.032	0.014	0.063	0.118	0.023	0.048
DBP	CrSO_2_	0.144	0.022	0.073	0.048	0.004	0.042	0.096	0.036
	CFTOE	0.137	0.001	0.058	0.061	0.017	0.076	0.023	0.004
MBP	CrSO_2_	0.109	0.008	0.116**	0571	0.000	0.024	0.044	0.008
	CFTOE	0.090	0.002	0.058	0.046	0.020	0.072	0.002	0.016

Results are expressed as *r*^2^ values.

LVO, left ventricular output; RVO, right ventricular output, MCA.MV, middle cerebral artery mean velocity; SBP, systolic blood pressure; DBP, diastolic blood pressure; MBP, mean blood pressure; SVC, superior vena cava; CFTOE, cerebral fractional tissue oxygen extraction; CrSO_2_, cerebral saturation.

Bold value indicate statistical significance.

* *p* = 0.047.

** *p* = 0.038.

## 4. Discussion

Our data suggests that during postnatal transition in ELGANs, despite major changes in pulmonary and systemic blood flow, there was no relationship detected between cerebral oxygenation and either echocardiography measures of systemic blood flow or BP for the range of measured values.

Previous literature on this subject is conflicting. In an earlier report, Kissack et al. reported that there was a negative correlation between CFTOE, measured after partial jugular venous obstruction, and LVO; however, this relationship was observed only in the presence of hypocarbia and when LVO was below the 5th percentile (5). Moran et al. reported a positive correlation between CrSO_2_ and SVC flow on postnatal day 1 in infants with birthweight <1,500 g (*n *= 27, *r* = 0.53) (7). Janaillac et al. reported that CrSO_2_ correlated with SVC flow in low cardiac output states during the first 72 h (*n *= 13, *r* = 0.74 at 6 h and *r *= 0.86 at 24 h) (9). Sortica Da Costa demonstrated that RVO and LVO correlated with CrSO_2_ at 24 h in infants with IVH (*n *= 13, *r *= 0.75) (10). On the other hand, several studies reported no correlation between either CFTOE (6) and CrSO_2_ (7) and RVO or LVO, or between CrSO_2_ and left ventricular ejection fraction (LVEF), Tricuspid annular plane systolic excursion (TAPSE) or SVC flow during transition (8, 11). Our findings are consistent with the latter observations (6–8, 11). The inconsistency between studies may relate to variance in patient demographics, illness severity, or developmental factors; however, a more plausible explanation is the fact that the interactions between systemic blood flow, arterial pressure and gestation-dependent changes are sufficiently complex and variable that binary correlative analyses may be overly simplistic. Unmeasured confounders include gestation-dependent vascular reactivity, and vascular compliance which may be influenced by arterial remodeling, and cellular metabolism.

Unlike other studies, we investigated the relationship between cerebral oxygenation and the components (systolic, and diastolic) of BP separately, rather than MBP alone. Pfurtscheller et al. studied the correlation between CrSO_2_ and MBP, DBP and SBP separately obtained by oscillometry in the first 15 min after birth in preterm neonates (16); however to our knowledge, such correlation over the 72 h after birth has not been reported before. Although most clinicians use MBP as an overall marker of cardiovascular health, with MBP < GA in mm Hg arbitrarily used as a definition of hypotension, it is now increasingly being recognized that this is a physiologic simplification. Studies of hypotension treatment show no apparent benefit when these thresholds are used (2). Biologically, SBP and DBP are considered to reflect stroke output and systemic vascular resistance, respectively. Therefore, some commentators have suggested treatment selection based on these subcomponents, although evidence of efficacy is limited (17, 18). In our cohort, we did not see any meaningful relationship between CrSO_2_ or CFTOE and SBP/DBP/MBP. A plausible explanation for the lack of relationship between cerebral oxygenation, and cardiac output and BP may be the presence of intact cerebral autoregulation. The finding of positive correlations between CrSO_2_ and SBP/MBP at a single time-point (17.3 h) is likely a chance finding. Alternatively, it may reflect fluctuations of cerebral autoregulation with periods of intact and impaired autoregulation (16). We also studied the correlation of cerebral oxygenation with MCA.MV as a more direct measure of cerebral blood flow, which, to our knowledge has not been studied before. However, we did not demonstrate a significant relationship with CrSO_2_ or CFTOE, which possibly reflects the role of cerebral microvasculature in autoregulation. It is also possible that the relationship between BP, cardiac output and cerebral blood flow is non-linear, multidirectional, and variable such that simple correlative analyses may not yield a meaningful pattern.

There are additional confounders that may impact the relationship between BP or cardiac output, and cerebral oxygenation. For example, during postnatal transition, significant left to right shunts across the PDA and PFO may increase pre-ductal LVO and RVO, respectively, further confounding the relationship between LVO and CrSO_2_. Even after excluding infants with a PDA measuring >1.5 mm and PFO > 2.0 mm, no correlation between LVO and RVO with CrSO_2_ and CFTOE at any time-point was noted. The lack of any relationship with measures of systemic blood flow may relate to the low number of patients with critically low cardiac output in this study.

The pattern of change over time in cerebral oxygenation and hemodynamic parameters presented in Table 2 is consistent with published literature (19–21). The increase in LVO and RVO is likely related to the change in loading conditions and myocardial adaptation over time (19). The change in cardiac output also is likely responsible for an increase in SBP seen in our cohort, but not DBP or MBP (20). Although the increase in CrSO_2_ was statistically significant, pairwise comparisons did not show any significant change compared to the baseline value. CFTOE, MCA.MV and SVC flow may likely be related to preserved cerebral autoregulation as SVC flow is representative of upper body and cerebral flow (21, 22).

Our data highlights the limitations of readily available hemodynamic indices that govern end-organ perfusion, to estimate cerebral blood flow. This is especially important in clinical decision making such as the management of transitional hypotension based on numerical values of BP. Although previous studies have shown a correlation between LVO and/or SVC flow and deranged cerebral neurophysiology in subgroups of neonates in individual studies (5, 9), we did not observe such a relationship. Therefore, longitudinal evaluation of cerebral oxygenation, together with other clinical markers or tissue perfusion, may guide clinical management in patients with a low cardiac output state.

We acknowledge the following important limitation; specifically, due to the small sample size and low number of infants who were clinically unstable hypotensive or had low LVO or SVC flow, correlation between cerebral and systemic hemodynamics in this high-risk population was not performed. Moreover, the levels of hemoglobin and carbon dioxide and administration of medications can also influence CrSO_2_. However, we were unable to account for them as the timing of bloodwork did not align with the specific neurophysiologic measurements. Further, prophylactic indomethacin may cause cerebral vasoconstriction and may potentially affect cerebral blood flow by modulating PDA shunt, which we were unable to account for (23). Further, in our study, we used time-point values to study correlation, which is one of the other limitations of our research. Time correlation measurements between mean blood pressure and CrSO_2_, are preferred for studying cerebral autoregulation (24). Therefore, objective measures of cerebral autoregulation are likely to be informative, especially in the critical management of sick and unstable neonates. Although the measurement of cerebral autoregulation in preterm infants is an emerging area of research (25), there is ongoing debate as to the most accurate algorithms and methods (24). The absence of bedside measures that estimate cerebral blood flow highlights an urgent need for robust studies aimed toward the translation of real-time continuous autoregulation measurement at the bedside as a part of clinical practice, especially for the sickest and most vulnerable preterm infants. Future research on this subject should focus on the most unstable neonates; in particular, an adequately powered study in infants with very low cardiac output, extreme hypotension or shock would the enable characterization of the relationship in the highest-risk patients while allowing to adjust for confounders would be of most value.

## Data Availability

The datasets presented in this article are not readily available. Requests to access the datasets should be directed to poorva.deshpande@sinaihealth.ca.
